# Development and Application of a Reverse-Transcription Recombinase-Aided Amplification Assay for Porcine Epidemic Diarrhea Virus

**DOI:** 10.3390/v14030591

**Published:** 2022-03-12

**Authors:** Xiuhong Wu, Yuanjia Liu, Liguo Gao, Zhuanqiang Yan, Qiqi Zhao, Feng Chen, Qingmei Xie, Xinheng Zhang

**Affiliations:** 1 Heyuan Branch, Guangdong Provincial Laboratory of Lingnan Modern Agricultural Science and Technology, College of Animal Science, South China Agricultural University, Guangzhou 510642, China; xhwu@stu.scau.edu.cn (X.W.); gaoliguo@stu.scau.edu.cn (L.G.); qiqi-zhao@stu.scau.edu.cn (Q.Z.); 31000350@scau.edu.cn (F.C.); 2 Guangdong Engineering Research Center for Vector Vaccine of Animal Virus, Guangzhou 510642, China; 3 South China Collaborative Innovation Center for Poultry Disease Control and Product Safety, Guangzhou 510642, China; 4 Guangdong Provincial Key Laboratory of Veterinary Pharmaceutics Development and Safety Evaluation, College of Veterinary Medicine, South China Agricultural University, Guangzhou 510642, China; andrewliu@stu.scau.edu.cn; 5 Guangdong Enterprise Key Laboratory for Animal Health and Environmental Control, Wen’s Foodstuff Group Co., Ltd., Yunfu 527439, China; aurora@stu.scau.edu.cn

**Keywords:** porcine epidemic diarrhea virus, reverse-transcription recombinase-aided amplification assay, constant temperature detection, clinical diagnosis

## Abstract

Porcine epidemic diarrhea virus (PEDV) is a coronavirus currently widespread worldwide in the swine industry. Since PEDV was discovered in China in 1984, it has caused huge economic losses in the swine industry. PEDV can infect pigs of all ages, but piglets have the highest infection with a death rate as high as 100%, and the clinical symptoms are watery diarrhea, vomiting, and dehydration. At present, there is not any report on PEDV detection by RT-RAA. In this study, we developed an isothermal amplification technology by using reverse-transcription recombinase-aided amplification assay (RT-RAA) combined with portable instruments to achieve a molecular diagnosis of PEDV in clinical samples from China. By designing a pair of RT-RAA primers and probes based on the PEDV N gene, this method breaks the limitations of existing detection methods. The assay time was within 30 min at 41 °C and can detect as few as 10 copies of PEDV DNA molecules per reaction. Sixty-two clinical tissue samples were detected by RT-qPCR and RT-RAA. The positive and negative rates for the two methods were 24.19% and 75.81%, respectively. Specificity assay showed that the RT-RAA had specifically detected PEDV and was not reactive for porcine parvovirus (PPV), transmissible gastroenteritis virus (TGEV), porcine circovirus type 2 (PCV2), porcine pseudorabies virus (PRV), porcine reproductive and respiratory syndrome virus (PRRSV), classical swine fever virus (CSFV), swine flu virus (SIV), or porcine Japanese encephalitis virus (JEV). The results suggested that RT-RAA had a strong specificity and high detection sensitivity when combined with a portable instrument to complete the detection under a constant temperature of 30 min, which are more suitable for preventing and controlling PEDV onsite in China.

## 1. Introduction

Porcine epidemic diarrhea virus (PEDV) is the pathogen of porcine epidemic diarrhea (PED), which can cause epidemic diseases in the global swine industry. PEDV is an enveloped virus, and a member of the Coronaviridae family and genus Alphacoronavirus. Its single- and positive-stranded RNA is similar to other coronaviruses, with a full-length genome of 28,033–28,038 nt [1]. The mainly clinical features of PED are acute watery diarrhea, vomiting, dehydration, and weight loss. Pigs of all ages are susceptible to PEDV, and the morbidity and mortality of suckling piglets reach 80–100%, causing huge economic losses in the swine industry [2,3]. Since PED was discovered in the UK in 1971, the disease has spread to European countries and Asian regions, especially China and South Korea [4,5,6]. In China, PEDV was first discovered on pig farms in 1984 [7,8]. The outbreak of PEDV was partly due to the rapid onset of the virus, the rapid spread of the virus, the high morbidity and mortality of piglets, and the widespread distribution [8,9]. In subsequent outbreaks of swine diarrhea disease, PED showed a high positive rate, which had a strong negative impact on the swine industry [10,11,12]. In December 2010, a new highly virulent strain of PEDV broke out in China that killed more than 1 million piglets, and the mortality rate for suckling piglets amounted to 100% [13,14]. In May 2013, PEDV suddenly appeared in the United States and was quickly distributed across the country and to Canada and Mexico. During the yearlong epidemic, more than 8 million newborn piglets were killed in the United States [15,16]. Subsequently, serious PED outbreaks occurred in Asian countries such as South Korea, China, and Japan [17,18,19]. In Lee’s report, he speculated that PEDV may be transmitted during transport or product input [20]. In China, in a research report containing an analysis of PEDV molecular structure, it was speculated that the PEDV prevalent in South Korea after 2013 may have come from China [21]. It was also indicated in other research reports that the trans-regional transmission of PEDV can be detected in vehicles or provender [22,23,24]. It was particularly necessary to choose a detection method with high sensitivity, strong specificity, a relatively low cost, and suitability for the scene, reducing the risk of PEDV transmission of regions.

In China, varieties of laboratory tools have been applied to detect and diagnose PEDV. Methods of detecting PEDV cover pathogen isolation, molecular methods, and immunoassays. Conventional molecular laboratory detection methods include conventional reverse transcription-polymerase chain reaction (RT-PCR) [25] and quantitative real-time reverse transcription PCR (RT-qPCR) [26]. These methods have high sensitivity and specificity, but they require well-trained experimenters, as well as expensive and sophisticated instruments. Furthermore, there were insurmountable disadvantages for onsite detection of the grassroots level. Loop-mediated isothermal amplification (LAMP) [27,28] requires 4–6 primers running at 60 °C for 1 h or a pair of primers running at 95 °C for more than 1 h [29,30,31]. Immunoassays such as the indirect fluorescent antibody (IFA) test [32,33] and an enzyme-linked immunosorbent assay (ELISA) [34,35] were conducted based on PEDV monoclonal antibodies [36]. However, the expression level of the antibodies can directly or indirectly affect the test results. Moreover, the IFA had a high professional quality of operators and the detection period was long. The detection sensitivities of both the IFA and ELISA were lower than the molecular detection line.

Another new isothermal amplification molecular diagnostic technology, reverse-transcription recombinase-aided amplification assay (RT-RAA), has the advantages of being faster, simple, and low-cost, and does not require class thermostable enzymes or complex thermal cycles. In recent years, it has been used for the agricultural detection of pathogens, and the method has shown specific and sensitive performance [37,38]. Tu reported on the application of swine fever virus and porcine pseudorabies virus in RT-RAA [39,40]. Wang reported on the detection of Newcastle disease virus and infectious bronchitis virus in chickens by RT-RAA [41,42]. In addition, Wang reported zoonosis of Toxoplasma gondii detection by RT-RAA [43]. Furthermore, RT-RAA also has been applied to human disease detection. For example, Qin reported application in the detection of the human norovirus GII.4 [44], and Xue and Zheng each reported the application in the detection of the novel SARS-CoV-2 virus [45,46]. However, until now, there has not been any report on PEDV detection by RT-RAA.

RT-RAA and reverse-transcription recombinase polymerase-aided amplification assay (RT-RPA) have similar amplification principles, but the source of the enzyme is different. The difference between the two is the root of the recombinase. The RPA recombinase was taken from Phage T4, and the RAA recombinase was derived from bacteria or fungus [47]. The RAA principle is that the recombinase is tightly combined with the primer to form a polymer of the enzyme and the primer at 35–42 °C. When the primers search for a completely matched complementary sequence on the template, with the help of the single-stranded DNA binding protein and DNA polymerase, a new complementary DNA strand is formed, and the amplified product grows exponentially. Thus, the RAA fluorescent probe works in conjunction with the exonuclease, allowing qualitative analysis of the results in real time.

The PEDV consists of seven open reading frames that encode four structural proteins and three nonstructural proteins; namely, the spike protein (S), membrane protein (M), envelope protein (E), nucleocapsid protein (N), replicases 1a and 1b, and ORF3 [48,49,50]. Some researchers reported that the S gene was appropriate for studying the genetic correlation of the virus [51]. In relevant studies, the S gene, ORF3, and nonstructural protein 2 (nsp2) and nsp3 were hypervariable regions [52]. Nevertheless, a previous study found that the PEDV N protein was the best candidate antigen for early diagnosis. Because the PEDV N gene was highly conserved [53], we designed the RT-RAA primers and probe on the PEDV N gene.

## 2. Materials and Methods

### 2.1. Virus and Clinical Specimens

The porcine parvovirus (PPV, batch no. 202104) and porcine circovirus type 2 (PCV2, batch no. 202102) were commercially available vaccines purchased from Harbin Pharmaceutical Group Bio-Vaccine Co., Ltd., (Harbin, China). The porcine pseudorabies virus (PRV, batch no. 20210388), classical swine fever virus (CSFV, batch no. 20210210) and swine flu virus (SIV, batch no. 20210101) were commercially available vaccines purchased from Wuhan Keqian Biology Co., Ltd., (Wuhan, China). The porcine Japanese encephalitis virus (JEV), porcine reproductive and respiratory syndrome virus (PRRSV), transmissible gastroenteritis virus (TGEV), and porcine epidemic diarrhea virus (PEDV) were provided by Wen’ s Foodstuff Group Co., Ltd., (Yunfu, China).

Sixty-two clinical samples in this study were provided by Wen’s Foodstuff Group Co., Ltd., (Yunfu, China). They were the intestinal tissues of pigs that died of diarrhea and were suspected of being infected with PEDV. They were stored at −80 °C in an ultralow-temperature freezer in animal health aquaculture with environmental control.

### 2.2. Reagents and Instruments

The Viral DNA/RNA isolation AxyPrep Body Fluid Viral DNA/RNA Miniprep Kit was purchased from Guangzhou Suyan Biotechnology Co., Ltd., (Guangzhou, China). The animal tissue total RNA extraction kit (DP431) was purchased from Guangzhou Sijia Biotechnology Co., Ltd., (Guangzhou, China). The real-time fluorescent RT-RAA reaction freeze-dried powder was purchased from Hangzhou ZC Bio-Sci & Tech Co., Ltd., (Hangzhou, China). The PEDV real-time fluorescent RT-PCR detection kit was purchased from Harbin Guosheng Biotechnology Co., Ltd., (Harbin, China). A real-time fluorescent quantitative PCR instrument (Bio-Rad Laboratories, model: CFX96, Shanghai, China) was used in this study.

### 2.3. Primers and Probe Design

**Figure 1:**
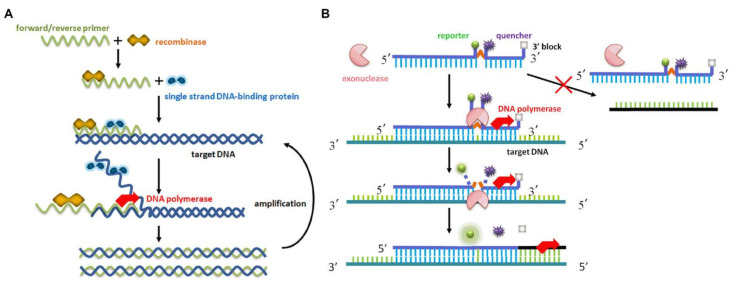
Schematic representation of the RAA-based amplification process: (**A**) reaction mechanism based on RAA recombination; (**B**) principle of real-time fluorescent RT-RAA.

The schematic diagram of the RAA method was previously reported by Fan [54], as shown in Figure 1. Referring to the PEDV N gene sequence in the GenBank database (GenBank accession numbers: JX188454, KJ020932, KJ646613, KC243782, JF690780, JF700126, JQ743654, JX406135, MG373547, MK458324, MK458327, KT799997, KY619826, KY619826, KY619825), we used DNAMAN (Lynnon Biosoft, version: v9.0, San Ramon, CA, USA) to align multiple sequences. According to gene comparison and analysis of conserved regions, the real-time fluorescent RT-RAA primers and probe (Table 1) were designed and synthesized by Sangon Biotech (Shanghai) Co., Ltd., (Shanghai, China).

### 2.4. Generation of Plasmid Standard

The standard plasmid of the PEDV N gene used in this study was synthesized by Sangon Biotech (Shanghai) Co., Ltd. (Shanghai, China). (GenBank accession number AJX188454.1: 26,376–27,701.)

### 2.5. Sample Preparation

We followed the recommended steps of the Viral DNA/RNA isolation AxyPrep Body Fluid Viral DNA/RNA Miniprep Kit to extract PPV, PCV2, PRV, PRRSV, CSFV, SIV, and JEV nucleic acid. Furthermore, the nucleic acid was stored at −80 °C in an ultralow-temperature freezer.

### 2.6. RT-RAA Primers Verification

Verifying the size of the designed primers, the positive control was the PEDV N gene plasmid, and the negative control was the ddH_2_O. The reaction system was as follows: F-Primer (10 µM) 1.0 µL, R-Primer (10 µM) 1.0 µL, ddH_2_O 6.0 µL, 2xEsTaq MasterMix 10.0 µL, template 2 µL. The reaction procedure was: 95 °C pre-denaturation for 3 min; 95 °C denaturation for 30 s, 58 °C renaturation for 30 s, 72 °C extension for 30 s, 35 cycles; 72 °C extension for 2 min. The obtained product was analyzed by 1% agarose gel electrophoresis.

### 2.7. Establishment and Optimization of RT-RAA Reaction System

Preparation for the real-time fluorescent RT-RAA reaction system: each test sample corresponded to the real-time fluorescent RT-RAA reaction dry powder tube. The reaction composition and volume of the real-time fluorescent RT-RAA reaction tube is shown in Table 2. After the reaction system was ready, we shook it gently to remove bubbles and centrifuged it at a low speed for 10 s. The amplification temperature of the real-time fluorescent RT-RAA reaction system was 41 °C, so the amplification program of the system was 41 °C for 40 s and 41 °C for 30 s for 40 cycles, and then the fluorescence signal was collected. After the reaction had completed, the positive control had a typical amplification curve, and a peak time ≤19.5 min (Ct value ≤ 39), which was a valid result; for the negative control, no amplification curve appeared, so the peak time >19.5 min (Ct value > 39) was a valid result.

### 2.8. Sensitivity Analysis of the Real-Time RT-RAA Assay

After diluting the PEDV-N plasmid by a 10-fold ratio, we used 5 concentration gradients as reactive templates, which were 10^5^ copies/µL, 10^4^ copies/µL, 10^3^ copies/µL, 10^2^ copies/µL, and 10^1^ copies/µL, and we took 2 µL of each concentration gradients as templates. Then, we repeated this three times to analyze the sensitivity assay. The real-time fluorescent RT-RAA amplification was carried out in the optimized reaction conditions shown above.

### 2.9. Specificity Analysis of the Real-Time RT-RAA Assay

We used the PEDV nucleic acid as a positive control and the ddH_2_O as a negative control. For nucleic acid of PPV, TGEV, PCV2, PRV, PRRSV, CSFV, SIV, and JEV, 2 µL of templates were respectively taken for specificity analysis and repeated three times. The real-time fluorescent RT-RAA amplification was carried out in the optimized reaction conditions shown above.

### 2.10. Repeatability Analysis of Real-Time RT-RAA Assay

In this study, three low-concentration gradients, including 10^3^ copies/µL, 10^2^ copies/µL, and 10^1^ copies/µL, were used as templates, and the volume of the reaction template for each concentration was 2 µL. We repeated this three times to analyze the repeatability assay. The real-time fluorescent RT-RAA amplification was carried out in the optimized reaction conditions shown above. 

### 2.11. Evaluation of the Real-Time RT-RAA Assay

Nucleic acids from 62 clinical samples were tested using the purchased real-time fluorescent RT-PCR detection kit and the real-time fluorescent RT-RAA method established in this study. The two detection results were used for comparative analysis to evaluate the established method in this study.

## 3. Results

### 3.1. RT-RAA Primer Verification

**Figure 2:**
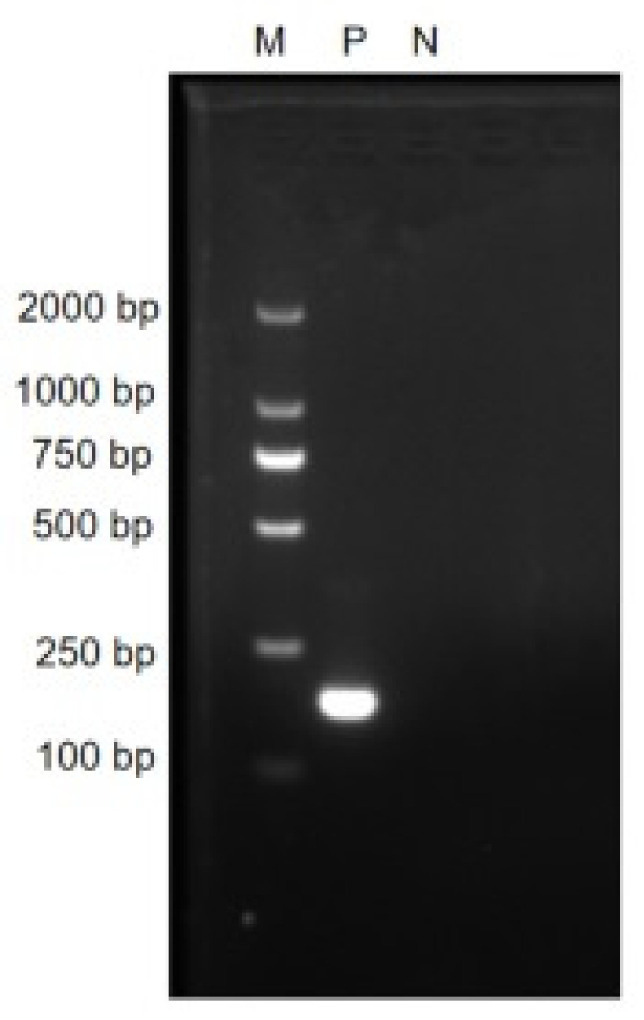
The agarose gel electrophoresis results of PCR amplified products with the real-time RT-RAA primers. M: D 2000 marker; P: positive control, i.e., PEDV N gene plasmid; N: negative control, i.e., ddH_2_O.

In order to verify the size of real-time fluorescent RT-RAA primer fragments, a PCR experiment was performed. The results are shown in Figure 2. The positive control had a target amplified fragment of a specific band with a size of 171 bp, which was our expected result. The negative control presented an absence of primer dimers and nonspecific bands. This showed that we designed real-time fluorescent RT-RAA primers that could specifically detect PEDV.

### 3.2. Establishment and Optimization of the Real-Time RT-RAA Reaction System

We optimized the real-time fluorescent RT-RAA reaction system and determined that the working concentrations of the primers and probes were both 10 µM. The best temperature for the real-time fluorescent RT-RAA reaction was 41 °C, and the reaction program was 41 °C for 40 s and 41 °C for 30 s for 40 cycles. The volume of the real-time fluorescent RT-RAA was presented in Table 2.

### 3.3. Sensitivity Analysis of the Real-Time RT-RAA Assay

**Figure 3:**
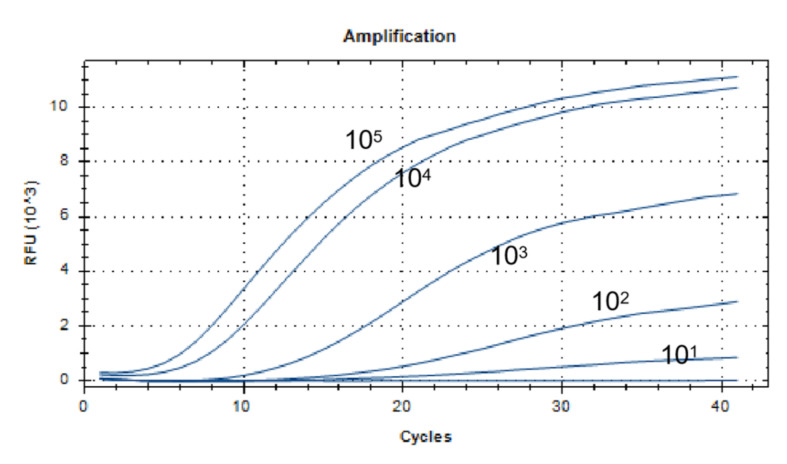
Sensitivity analysis of the real-time RT-RAA assay. The dilution range of the PEDV-N gene plasmid was 10^1^ to 10^5^ copies/µL, and the lowest detection line was 10^1^ copies/µL.

The lowest detection line for the real-time fluorescent RT-RAA primers and probes designed in this study was 10^1^ copies/µL. We selected a representative result of the three independent experiments as shown in Figure 3.

### 3.4. Specificity Analysis of the Real-Time RT-RAA Assay

**Figure 4:**
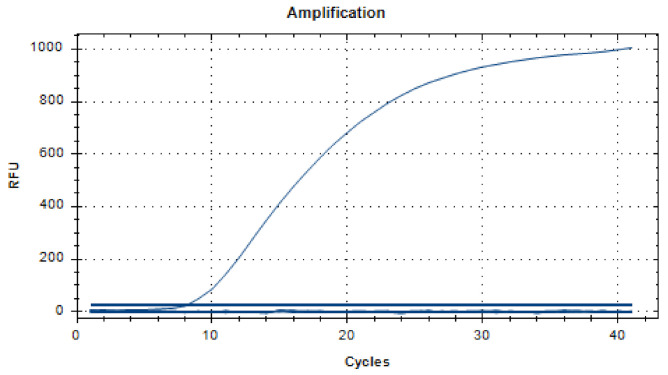
Specificity analysis of the real-time RT-RAA assay. The RT-RAA method specifically detected PEDV nucleic acid, and had no cross-reactivity with porcine parvovirus (PPV), transmissible gastroenteritis virus (TGEV), porcine circovirus type 2 (PCV2), porcine pseudorabies virus (PRV), porcine reproductive and respiratory syndrome virus (PRRSV), classical swine fever virus (CSFV), swine flu virus (SIV), or porcine Japanese encephalitis virus (JEV) nucleic acid. The figure is a representative result of the three independent experiments.

As shown in Figure 4, the positive control had a fluorescent signal, and the negative control and other viruses had no fluorescent signal. This showed that the established real-time fluorescent RT-RAA method could better distinguish PEDV from PPV, TGEV, PCV2, PRV, PRRSV, CSFV, SIV and JEV, and specifically detected PEDV.

### 3.5. Repeatability Analysis of Real-Time RT-RAA Assay

**Figure 5:**
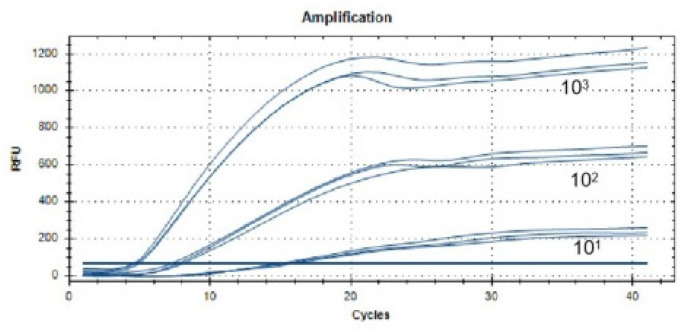
Repeatability analysis of the real-time RT-RAA assay. The real-time RT-RAA assay was repeated three times with 10^3^ copies/µL, 10^2^ copies/µL, and 10^1^ copies/µL.

As presented in Figure 5, the results showed that the assays repeated three times with 10^3^ copies/µL, 10^2^ copies/µL, and 10^1^ copies/µL were consistent, including the amplification reaction peak time and the curve shape. The coefficients of variation (CVs) were 2.28%, 5.23%, and 5.29%, and they were all within 10% (as shown in Table 3), which meant that we established a real-time fluorescent RT-RAA method that had good repeatability.

### 3.6. Evaluation of the Real-Time RT-RAA Assay

The detection results are shown in Table 4. We compared the detection results of the PEDV RT-qPCR and the real-time fluorescent RT-RAA, and we found that the positive and negative results of the two methods were identical. The positive and negative rates for the two methods were 24.19% and 75.81%, respectively. We also compared the times for detection; PEDV RT-qPCR took more than 90 min, but the real-time fluorescent RT-RAA only needed 30 min, which meant that compared with RT-qPCR, the RT-RAA assay could not only obtain accurate results quickly, but also was more portable and could save more time.

## 4. Discussion

In China, prevention and control of PEDV is based primarily on vaccines, including inactivated vaccines, attenuated live vaccines, and subunit vaccines [55,56,57,58,59]. Moreover, there are high expectations for nucleic acid vaccines with a low cost, high degree of safety, short lead time, and simple design [60], but nucleic acid vaccines, including DNA vaccines and mRNA vaccines, are still under development [61,62]. However, the latest progress of research on PEDV noted that because there was no effective vaccine currently available, PEDV still poses a huge threat to the pig industry around the world [63]. For example, since 2014, PEDV has caused severe outbreaks in some states in the United States [64,65]. Whether the vaccines are effective or not, PEDV can be fatal in a herd of unhealthy pigs or piglets with weakened immunity. Therefore, rapid, accurate, low-cost, and clinically suitable diagnostic methods are necessary for the prevention and control of PEDV. The PEDV N gene sequence is highly conserved, and the PEDV N protein can be detected in the early stage of infection [54], so we selected the PEDV N gene as the target.

For the detection of PEDV in China, the common molecular biological methods were used, including RT-PCR, RT-qPCR, and LAMP, because these methods provided high sensitivity, and specific and accurate results. However, these methods were applied in the laboratory rather than in onsite detection. PEDV had a high risk of transmission during transportation, including factors such as: transport trailers, farm workers’ hands, boots and clothes, feed ingredients, and additives [66,67,68,69]. PEDV cross-contamination also took place during feed production, and it remained infectious on handbag materials for 35 days at room temperature [68,70]. Furthermore, PEDV can infect nursing pigs up to 10 miles away by forming aerosol particles [71,72]. Therefore, the method of onsite detection is inevitably a way to overcome this dilemma. The RT-RAA is a thermostatic amplification detection method that combines probes to accurately determine the target fragment and returns accurate results within 30 min. We used the RT-RAA reagent from Hangzhou ZC Bio-Sci & Tech Co., Ltd. (Hangzhou, China). The company produces a portable real-time fluorescence detection instrument that weighs 1.75 kg and has a lithium polymer battery so that it can work in an onsite environment without a power supply for a long time, which is convenient to use in when there are limited resources onsite.

We found that Jiang established an RT-PCR sensitivity of 10^4^ copies/µL [73], and Pan established an RT-qPCR sensitivity of 10^2^ copies/µL [74]. In this study, the sensitivity of the RT-RAA was 10^1^ copies/µL, which was 1000 times for RT-PCR and 10 times for RT-qPCR. The sensitivity was the same as in Yu’s report on LAMP of 10^1^ copies/µL [28], but the RT-RAA amplified fragments were smaller, and the combination of protein and probe was more conducive to identifying target fragments. Thus, the probability of false positives for the RT-RAA was smaller. We also verified the specificity of the RT-RAA’s primers and probes, and the results showed that it only specifically detected PEDV and did not cross-react with PPV, TGEV, PCV2, PRV, PRRSV, CSFV, SIV, or JEV. The repeatability assay of three low-concentration templates with the CV had results of 2.28%, 5.23%, 5.29%, and they were all within 10%, indicating that the RT-RAA method had excellent repeatability.

In addition, the newly developed RT-RAA method was compared with the commercial PEDV RT-qPCR, and we found that the coincidence rate of both the positive and negative controls was 100%. The positive rate was 24.19%, and the negative rate was 75.81%. Our results demonstrated a high correlation between the RT-RAA and commercial RT-qPCR. Importantly, the RT-RAA detection time was within 30 min, and the test results could be seen in 5 min at the fastest, saving at least 1 h over RT-qPCR. This is crucial to the minute-by-minute battle in pandemic surveillance.

## 5. Conclusions

We successfully established an RT-RAA method to detect PEDV onsite within 30 min. The RT-RAA had the advantages of high sensitivity, strong specificity, good repeatability, and low cost, which can be key factors in the prevention and control of PEDV in China.

## Figures and Tables

**Table 1:** Sequences of primers and probes for real-time RT-RAA assay.

Primer/Probe	Sequence (5′–3′)	Gen Localization
F-Primer	AATCGTGGAAATAACCAGGGTCGTGGAG	26904–26931
R-Primer	CAAAGATTTAAGGGCATCCTTGACAGCAG	27046–27074
P-Probe	AACAGAGGAGGCAATAATAATAACAATAACAAG /i6FAMdT//THF//iBHQ1dT/CGTAACCAGTCCAAG (C3-spacer)	26940–26990

**Table 2:** The real-time RT-RAA reaction system preparation table.

RT-RAA Reaction System	Usage
RT-RAA freeze-dried powder	1 tube
A buffer	25 µL
B buffer	2.5 µL
F-Primer (10µM)	2 µL
R-Primer (10µM)	2 µL
P-Probe (10µM)	0.6 µL
H_2_O	15.9 µL
RNA	2 µL
Total	50 µL

**Table 3:** Data from the repeatability analysis of the real-time RT-RAA assay.

Templates (Copies/µL)	1	2	3	Average ± SD	CV%
10^1^	15.01	15.34	15.71	15.35 ± 0.35	2.28
10^2^	7.69	8.05	7.25	7.66 ± 0.40	5.23
10^3^	4.99	4.47	4.66	4.71 ± 0.26	5.59

**Table 4:** The results of clinical tests.

Methods and Determination Indexes	Positive Tissue Numbers	Negative Tissue Numbers	Total
Commercial RT-qPCR	15	47	62
RT-RAA	15	47	62
Positive rate (%)	24.19	-	-
Negative rate (%)	-	75.81	-
Coincidence rate (%)	100	100	-

## Data Availability

All data used and presented in this study were either available in public repositories as described in the Materials and Methods section or were made available in the NCBI database.

## References

[1] Wang P., Zhu J., Liu X., Guo J., Gu X., Ruan W. (2019). Isolation and recombinant analysis of variants of porcine epidemic diarrhea virus strains from Beijing, China. VirusDisease.

[2] Li B., Du L., Yu Z., Sun B., Xu X., Fan B., Guo R., Yuan W., He K. (2017). Poly (d,l-lactide-co-glycolide) nanoparticle-entrapped vaccine induces a protective immune response against porcine epidemic diarrhea virus infection in piglets. Vaccine.

[3] Beall A., Yount B., Lin C.M., Hou Y., Wang Q., Saif L., Baric R. (2016). Characterization of a Pathogenic Full-Length cDNA Clone and Transmission Model for Porcine Epidemic Diarrhea Virus Strain PC22A. mBio.

[4] Park S.J., Song D.S., Park B.K. (2013). Molecular epidemiology and phylogenetic analysis of porcine epidemic diarrhea virus (PEDV) field isolates in Korea. Arch. Virol..

[5] Diep N.V., Sueyoshi M., Izzati U., Fuke N., Teh A.P.P., Lan N.T., Yamaguchi R. (2018). Appearance of US-like porcine epidemic diarrhoea virus (PEDV) strains before US outbreaks and genetic heterogeneity of PEDVs collected in Northern Vietnam during 2012–2015. Transbound. Emerg. Dis..

[6] Wood E.N. (1977). An apparently new syndrome of porcine epidemic diarrhoea. Vet. Rec..

[7] Bi J., Zeng S., Xiao S., Chen H., Fang L. (2012). Complete genome sequence of porcine epidemic diarrhea virus strain AJ1102 isolated from a suckling piglet with acute diarrhea in China. J. Virol..

[8] Pan Y., Tian X., Li W., Zhou Q., Wang D., Bi Y., Chen F., Song Y. (2012). Isolation and characterization of a variant porcine epidemic diarrhea virus in China. Virol. J..

[9] Li Z., Chen F., Ye S., Guo X., Muhanmmad Memon A., Wu M., He Q. (2016). Comparative Proteome Analysis of Porcine Jejunum Tissues in Response to a Virulent Strain of Porcine Epidemic Diarrhea Virus and Its Attenuated Strain. Viruses.

[10] Yang D., Su M., Li C., Zhang B., Qi S., Sun D., Yin B. (2020). Isolation and characterization of a variant subgroup GII-a porcine epidemic diarrhea virus strain in China. Microb. Pathog..

[11] Zhang H., Xia M., Ju D., Wu B., Ning C., Song N., Feng T., Chen F., Wang X., Wu Y. (2017). Isolation, molecular characterization and an artificial infection model for a variant porcine epidemic diarrhea virus strain from Jiangsu Province, China. Arch. Virol..

[12] Gao X., Li D., Zhao J., Xu F., Ge X., Guo X., Han J., Yang H., Zhou L. (2016). Complete Genome Sequence of Porcine Epidemic Diarrhea Virus from an Outbreak in a Vaccinated Farm in Shandong, China. Genome Announc..

[13] Li W., Li H., Liu Y., Pan Y., Deng F., Song Y., Tang X., He Q. (2012). New variants of porcine epidemic diarrhea virus, China, 2011. Emerg. Infect. Dis..

[14] Zhang L.P., Liu X.S., Zhang Q.L., Zhou P., Fang Y.Z., Dong Z.L., Zhao D.H., Li W.Y., Feng J.X., Zhang Y.G. (2019). Biological characterization and pathogenicity of a newly isolated Chinese highly virulent genotype GIIa porcine epidemic diarrhea virus strain. Arch. Virol..

[15] Ojkic D., Hazlett M., Fairles J., Marom A., Slavic D., Maxie G., Alexandersen S., Pasick J., Alsop J., Burlatschenko S. (2015). The first case of porcine epidemic diarrhea in Canada. Can. Vet. J..

[16] Stevenson G.W., Hoang H., Schwartz K.J., Burrough E.R., Sun D., Madson D., Cooper V.L., Pillatzki A., Gauger P., Schmitt B.J. (2013). Emergence of Porcine epidemic diarrhea virus in the United States: Clinical signs, lesions, and viral genomic sequences. J. Vet. Diagn. Investig..

[17] Lin C.N., Chung W.B., Chang S.W., Wen C.C., Liu H., Chien C.H., Chiou M.T. (2014). US-like strain of porcine epidemic diarrhea virus outbreaks in Taiwan, 2013–2014. J. Vet. Med. Sci..

[18] Furutani A., Sekiguchi S., Sueyoshi M., Sasaki Y. (2019). Effect of intervention practices to control the porcine epidemic diarrhea (PED) outbreak during the first epidemic year (2013-2014) on time to absence of clinical signs and the number of dead piglets per sow in Japan. Prev. Vet. Med..

[19] Lee S., Lee C. (2014). Outbreak-Related Porcine Epidemic Diarrhea Virus Strains Similar to US Strains, South Korea, 2013. Emerg. Infect. Dis..

[20] Lee C. (2016). Erratum to: Porcine epidemic diarrhea virus: An emerging and re-emerging epizootic swine virus. Virol. J..

[21] Sun M., Ma J., Wang Y., Wang M., Song W., Zhang W., Lu C., Yao H. (2015). Genomic and epidemiological characteristics provide new insights into the phylogeographical and spatiotemporal spread of porcine epidemic diarrhea virus in Asia. J. Clin. Microbiol..

[22] Lowe J., Gauger P., Harmon K., Zhang J., Connor J., Yeske P., Loula T., Levis I., Dufresne L., Main R. (2014). Role of transportation in spread of porcine epidemic diarrhea virus infection, United States. Emerg. Infect. Dis..

[23] Dee S., Clement T., Schelkopf A., Nerem J., Knudsen D., Christopher-Hennings J., Nelson E. (2014). An evaluation of contaminated complete feed as a vehicle for porcine epidemic diarrhea virus infection of naïve pigs following consumption via natural feeding behavior: Proof of concept. BMC Vet. Res..

[24] Pasick J., Berhane Y., Ojkic D., Maxie G., Embury-Hyatt C., Swekla K., Handel K., Fairles J., Alexandersen S. (2014). Investigation into the role of potentially contaminated feed as a source of the first-detected outbreaks of porcine epidemic diarrhea in Canada. Transbound. Emerg. Dis..

[25] Song D.S., Kang B.K., Oh J.S., Ha G.W., Yang J.S., Moon H.J., Jang Y.S., Park B.K. (2006). Multiplex reverse transcription-PCR for rapid differential detection of porcine epidemic diarrhea virus, transmissible gastroenteritis virus, and porcine group A rotavirus. J. Vet. Diagn. Investig..

[26] Zhou X., Zhang T., Song D., Huang T., Peng Q., Chen Y., Li A., Zhang F., Wu Q., Ye Y. (2017). Comparison and evaluation of conventional RT-PCR, SYBR green I and TaqMan real-time RT-PCR assays for the detection of porcine epidemic diarrhea virus. Mol. Cell. Probes.

[27] Qiu X., Li T., Zhang G., Cao J., Jin Y., Xing G., Liao M., Zhou J. (2012). Development of a loop-mediated isothermal amplification method to rapidly detect porcine circovirus genotypes 2a and 2b. Virol. J..

[28] Yu X., Shi L., Lv X., Yao W., Cao M., Yu H., Wang X., Zheng S. (2015). Development of a real-time reverse transcription loop-mediated isothermal amplification method for the rapid detection of porcine epidemic diarrhea virus. Virol. J..

[29] Notomi T., Mori Y., Tomita N., Kanda H. (2015). Loop-mediated isothermal amplification (LAMP): Principle, features, and future prospects. J. Microbiol..

[30] Miller L.C., Crawford K.K., Lager K.M., Kellner S.G., Brockmeier S.L. (2016). Evaluation of two real-time polymerase chain reaction assays for Porcine epidemic diarrhea virus (PEDV) to assess PEDV transmission in growing pigs. J. Vet. Diagn. Investig..

[31] Ren X., Li P. (2011). Development of reverse transcription loop-mediated isothermal amplification for rapid detection of porcine epidemic diarrhea virus. Virus Genes.

[32] Okda F., Lawson S., Liu X., Singrey A., Clement T., Hain K., Nelson J., Christopher-Hennings J., Nelson E.A. (2016). Development of monoclonal antibodies and serological assays including indirect ELISA and fluorescent microsphere immunoassays for diagnosis of porcine deltacoronavirus. BMC Vet. Res..

[33] Okda F., Liu X., Singrey A., Clement T., Nelson J., Christopher-Hennings J., Nelson E.A., Lawson S. (2015). Development of an indirect ELISA, blocking ELISA, fluorescent microsphere immunoassay and fluorescent focus neutralization assay for serologic evaluation of exposure to North American strains of Porcine Epidemic Diarrhea Virus. BMC Vet. Res..

[34] Lin H., Zhou H., Gao L., Li B., He K., Fan H. (2018). Development and application of an indirect ELISA for the detection of antibodies to porcine epidemic diarrhea virus based on a recombinant spike protein. BMC Vet. Res..

[35] Fan J.H., Zuo Y.Z., Shen X.Q., Gu W.Y., Di J.M. (2015). Development of an enzyme-linked immunosorbent assay for the monitoring and surveillance of antibodies to porcine epidemic diarrhea virus based on a recombinant membrane protein. J. Virol. Methods.

[36] Sozzi E., Moreno A., Lelli D., Perulli S., Prosperi A., Brocchi E., Capucci L., Papetti A., Giacomini E., Alborali G.L. (2018). Development and validation of a monoclonal antibody-based competitive ELISA for detection of antibodies against porcine epidemic diarrhoea virus (PEDV). Res. Vet. Sci..

[37] Wang Z.H., Li P., Lin X., Jia H., Jiang Y.T., Wang X.J., Hou S.H. (2021). Application of portable real-time recombinase-aided amplification (rt-RAA) assay in the clinical diagnosis of ASFV and prospective DIVA diagnosis. Appl. Microbiol. Biotechnol..

[38] Wang Q.Y., Li F., Shen X.X., Fu S.H., He Y., Lei W.W., Liang G.D., Wang H.Y., Ma X.J. (2019). A Reverse-transcription Recombinase-aided Amplification Assay for the Rapid Detection of the Far-Eastern Subtype of Tick-borne Encephalitis Virus. Biomed. Environ. Sci..

[39] Tu F., Yang X., Xu S., Chen D., Zhou L., Ge X., Han J., Zhang Y., Guo X., Yang H. (2021). Development of a fluorescent probe-based real-time reverse transcription recombinase-aided amplification assay for the rapid detection of classical swine fever virus. Transbound. Emerg. Dis..

[40] Tu F., Zhang Y., Xu S., Yang X., Zhou L., Ge X., Han J., Guo X., Yang H. (2021). Detection of pseudorabies virus with a real-time recombinase-aided amplification assay. Transbound. Emerg. Dis..

[41] Wang W., Wang C., Zhang P., Yao S., Liu J., Zhai X., Zhang T. (2020). Reverse transcription recombinase-aided amplification assay combined with a lateral flow dipstick for detection of avian infectious bronchitis virus. Poult. Sci..

[42] Wang W., Wang C., Bai Y., Zhang P., Yao S., Liu J., Zhang T. (2020). Establishment of reverse transcription recombinase-aided amplification-lateral-flow dipstick and real-time fluorescence-based reverse transcription recombinase-aided amplification methods for detection of the Newcastle disease virus in chickens. Poult. Sci..

[43] Wang Z.H., Zhang W., Zhang X.Z., Yao X.R., Huang W., Jia H., Liu X.L., Hou S.H., Wang X.J. (2021). Development of a real-time recombinase-aided amplification (RT-RAA) molecular diagnosis assay for sensitive and rapid detection of Toxoplasma gondii. Vet. Parasitol..

[44] Qin Z., Xue L., Cai W., Gao J., Jiang Y., Yang J., Liang Y., Wang L., Zhang J., Hu Y. (2021). Development of a recombinase-aided amplification assay for rapid detection of human norovirus GII.4. BMC Infect. Dis..

[45] Xue G., Li S., Zhang W., Du B., Cui J., Yan C., Huang L., Chen L., Zhao L., Sun Y. (2020). Reverse-Transcription Recombinase-Aided Amplification Assay for Rapid Detection of the 2019 Novel Coronavirus (SARS-CoV-2). Anal. Chem..

[46] Zheng Y.Z., Chen J.T., Li J., Wu X.J., Wen J.Z., Liu X.Z., Lin L.Y., Liang X.Y., Huang H.Y., Zha G.C. (2021). Reverse Transcription Recombinase-Aided Amplification Assay with Lateral Flow Dipstick Assay for Rapid Detection of 2019 Novel Coronavirus. Front. Cell. Infect. Microbiol..

[47] Lü B., Cheng H.R., Yan Q.F., Huang Z.J., Shen G.F., Zhang Z.F., Li Y.N., Deng Z.X., Lin M., Cheng Q. (2010). Recombinase-aid amplification: A novel technology of in vitro rapid nucleic acid amplification. Sci. Sin. Vitae.

[48] Bridgen A., Kocherhans R., Tobler K., Carvajal A., Ackermann M. (1998). Further analysis of the genome of porcine epidemic diarrhoea virus. Adv. Exp. Med. Biol..

[49] Duarte M., Tobler K., Bridgen A., Rasschaert D., Ackermann M., Laude H. (1994). Sequence analysis of the porcine epidemic diarrhea virus genome between the nucleocapsid and spike protein genes reveals a polymorphic ORF. Virology.

[50] Kocherhans R., Bridgen A., Ackermann M., Tobler K. (2001). Completion of the porcine epidemic diarrhoea coronavirus (PEDV) genome sequence. Virus Genes.

[51] Lee D.K., Park C.K., Kim S.H., Lee C. (2010). Heterogeneity in spike protein genes of porcine epidemic diarrhea viruses isolated in Korea. Virus Res..

[52] Su M., Li C., Qi S., Yang D., Jiang N., Yin B., Guo D., Kong F., Yuan D., Feng L. (2020). A molecular epidemiological investigation of PEDV in China: Characterization of co-infection and genetic diversity of S1-based genes. Transbound. Emerg. Dis..

[53] Diel D.G., Lawson S., Okda F., Singrey A., Clement T., Fernandes M.H.V., Christopher-Hennings J., Nelson E.A. (2016). Porcine epidemic diarrhea virus: An overview of current virological and serological diagnostic methods. Virus Res..

[54] Fan X., Li L., Zhao Y., Liu Y., Liu C., Wang Q., Dong Y., Wang S., Chi T., Song F. (2020). Clinical Validation of Two Recombinase-Based Isothermal Amplification Assays (RPA/RAA) for the Rapid Detection of African Swine Fever Virus. Front. Microbiol..

[55] Yap Y.K., Smith D.R. (2010). Strategies for the plant-based expression of dengue subunit vaccines. Biotechnol. Appl. Biochem..

[56] Baek P.S., Choi H.W., Lee S., Yoon I.J., Lee Y.J., Lee S., Lee C. (2016). Efficacy of an inactivated genotype 2b porcine epidemic diarrhea virus vaccine in neonatal piglets. Vet. Immunol. Immunopathol..

[57] DeZure A.D., Berkowitz N.M., Graham B.S., Ledgerwood J.E. (2016). Whole-Inactivated and Virus-Like Particle Vaccine Strategies for Chikungunya Virus. J. Infect. Dis..

[58] Lauring A.S., Jones J.O., Andino R. (2010). Rationalizing the development of live attenuated virus vaccines. Nat. Biotechnol..

[59] Li L., Petrovsky N. (2016). Molecular mechanisms for enhanced DNA vaccine immunogenicity. Expert Rev. Vaccines.

[60] Pardi N., Hogan M.J., Porter F.W., Weissman D. (2018). mRNA vaccines—A new era in vaccinology. Nat. Rev. Drug Discov..

[61] Zhang Y., Zhang X., Liao X., Huang X., Cao S., Wen X., Wen Y., Wu R., Liu W. (2016). Construction of a bivalent DNA vaccine co-expressing S genes of transmissible gastroenteritis virus and porcine epidemic diarrhea virus delivered by attenuated Salmonella typhimurium. Virus Genes.

[62] Naik R., Peden K. (2020). Regulatory Considerations on the Development of mRNA Vaccines. Current Topics in Microbiology and Immunology.

[63] Jung K., Saif L.J., Wang Q. (2020). Porcine epidemic diarrhea virus (PEDV): An update on etiology, transmission, pathogenesis, and prevention and control. Virus Res..

[64] Ma Y., Zhang Y., Liang X., Lou F., Oglesbee M., Krakowka S., Li J. (2015). Origin, evolution, and virulence of porcine deltacoronaviruses in the United States. mBio.

[65] Wang L., Byrum B., Zhang Y. (2014). Detection and genetic characterization of deltacoronavirus in pigs, Ohio, USA, 2014. Emerg. Infect. Dis..

[66] Gordon R.K., Kotowski I.K., Coulson K.F., Link D., MacKenzie A., Bowling-Heyward J. (2019). The Role of Non-animal Origin Feed Ingredients in Transmission of Viral Pathogens of Swine: A Review of Scientific Literature. Front. Vet. Sci..

[67] Niederwerder M.C., Hesse R.A. (2018). Swine enteric coronavirus disease: A review of 4 years with porcine epidemic diarrhoea virus and porcine deltacoronavirus in the United States and Canada. Transbound. Emerg. Dis..

[68] Schumacher L.L., Cochrane R.A., Huss A.R., Gebhardt J.T., Woodworth J.C., Stark C.R., Jones C.K., Bai J., Main R.G., Chen Q. (2018). Feed batch sequencing to decrease the risk of porcine epidemic diarrhea virus (PEDV) cross-contamination during feed manufacturing. J. Anim. Sci..

[69] Bowman A.S., Krogwold R.A., Price T., Davis M., Moeller S.J. (2015). Investigating the introduction of porcine epidemic diarrhea virus into an Ohio swine operation. BMC Vet. Res..

[70] Scott A., McCluskey B., Brown-Reid M., Grear D., Pitcher P., Ramos G., Spencer D., Singrey A. (2016). Porcine epidemic diarrhea virus introduction into the United States: Root cause investigation. Prev. Vet. Med..

[71] Li Y., Wu Q., Huang L., Yuan C., Wang J., Yang Q. (2018). An alternative pathway of enteric PEDV dissemination from nasal cavity to intestinal mucosa in swine. Nat. Commun..

[72] Gallien S., Andraud M., Moro A., Lediguerher G., Morin N., Gauger P.C., Bigault L., Paboeuf F., Berri M., Rose N. (2018). Better horizontal transmission of a US non-InDel strain compared with a French InDel strain of porcine epidemic diarrhoea virus. Transbound. Emerg. Dis..

[73] Jiang C., He H., Zhang C., Zhang X., Han J., Zhang H., Luo Y., Wu Y., Wang Y., Ge B. (2019). One-step triplex reverse-transcription PCR detection of porcine epidemic diarrhea virus, porcine sapelovirus, and porcine sapovirus. J. Vet. Diagn. Investig..

[74] Pan Z., Lu J., Wang N., He W.T., Zhang L., Zhao W., Su S. (2020). Development of a TaqMan-probe-based multiplex real-time PCR for the simultaneous detection of emerging and reemerging swine coronaviruses. Virulence.

